# Retraction: An Occult Hepatitis B-Derived Hepatoma Cell Line Carrying Persistent Nuclear Viral DNA and Permissive for Exogenous Hepatitis B Virus Infection

**DOI:** 10.1371/journal.pone.0246549

**Published:** 2021-01-28

**Authors:** The PLOS ONE Editors

Following the publication of this article [1], concerns were raised regarding results presented in Fig 5. Specifically:

- Vertical irregularities suggestive of image splicing have been detected between lanes 10–11, 17–18, 18–19, and 20-P in Fig 5A and between lanes 3–4, 5–6, 10–11, 12–13, 15–16, and 17–18 in Fig 5B. The authors explained that the panels were spliced after additional data points were added to the original figure at the reviewer’s request.
- In Fig 5A, the results shown in lanes 11–12 appear to partially overlap with those shown in lanes 17–18 and 19–20 despite representing different experimental conditions.
- In Fig 5B, the results shown in lanes C-1 appear to partially overlap with those shown in lanes 3–5 despite representing different experimental conditions.
- The authors have provided underlying blots for the panels presented in Fig 5A and Fig 5B, and although these blots confirm the image splicing, they do not clarify the similarity within the panels discussed above. Furthermore, the underlying data provided for lanes 9, 10, 18 and 19 of Fig 5A and lanes C-7, 11, 12, 16, and 17 of Fig 5B did not match the results presented in the published panels.

In light of the concerns affecting multiple figure panels that question the integrity of these data, the *PLOS ONE* Editors retract this article.

CTY agreed with the retraction. CLL, RNC, SHM, PYK, and CCL either did not respond directly or could not be reached.
